# ECT in an Adolescent With Schizophrenia and Seizures: Case Report

**DOI:** 10.3389/fpsyt.2021.646466

**Published:** 2021-11-12

**Authors:** Anna Gralewicz, Łukasz Świȩcicki, Anna Z. Antosik-Wójcińska, Magdalena Konopko, Iwona Kurkowska-Jastrzȩbska, Halina Sienkiewicz-Jarosz, Łukasz Szostakiewicz, Barbara Remberk

**Affiliations:** ^1^Child and Adolescent Psychiatry Department, Institute of Psychiatry and Neurology (IPiN), Warsaw, Poland; ^2^Second Psychiatry Department, Institute of Psychiatry and Neurology (IPiN), Warsaw, Poland; ^3^Department of Psychiatry, Medical University of Warsaw, Warsaw, Poland; ^4^First Neurology Department, Institute of Psychiatry and Neurology (IPiN), Warsaw, Poland; ^5^Second Neurology Department, Institute of Psychiatry and Neurology (IPiN), Warsaw, Poland

**Keywords:** ECT, seizure, early onset schizophrenia (EOS), adolescent, case report

## Abstract

Electroconvulsive therapy (ECT) has been recognized as an effective treatment option in catatonia, and for prolonged or severe affective episodes and schizophrenia. Response rates vary from 40 to 80% in adolescents. The procedure is safe if the required precautions are undertaken. Nonetheless, ECT remains a serious clinical challenge in patients with comorbid seizures. We present a case study of a 17-year-old female student suffering from schizophrenia who was scheduled for ECT due to prior treatment inefficacy. Seizures had occurred a few days before the first ECT session. Nevertheless, the patient received the ECT course, combined with clozapine at 125 mg/day, after neurological diagnosis and treatment modification because the illness became life-threatening. The patient's clinical outcome was satisfactory without any seriously adverse events and further improvements were observed in the mental state following long-term psychosocial treatment at our inpatient unit. A few months later, epilepsy was however diagnosed with probably coexistence of partial seizures and seizure-like events without EEG correlate. Administering ECT in patients with seizure comorbidity was also investigated based on previous research. Data on this is, however, extremely scarce and to the best of our knowledge, the safety and efficacy of using ECT in adolescents with schizophrenia and seizures has yet not to any great extent been discussed in the literature.

## Introduction

Electroconvulsive therapy (ECT) is recognized as an effective treatment option for catatonia and in cases of treatment-refractory or severe manic episodes and schizophrenia. The National Institute for Health and Care Excellence recommends this as an alternative for acute treatment of life-threatening severe depression whenever a rapid response is required, or when other treatments have failed (1). The efficacy of ECT has recently been proven in numerous studies with the response rates ranging from 60 to 80% (2). A retrospective study by the Mayo Clinic found ECT response rates in an adolescent population to be 80% for catatonia, 80% for mania, 63% for depression, and 42% for schizophrenia (3). This method however seems to be underutilized in this population group (4).

### Aim of the Study

We present a case study of a 17-year-old patient with treatment-resistant, early-onset schizophrenia who was qualified for ECT therapy. Seizures occurred during the preparations for clinic. Due to the patient's life-threatening mental state, the ECT protocol was completed with a favorable outcome, despite the risk of seizures after neurological diagnosis and treatment. The association between ECT and seizures is discussed, along with their clinical implications. The patient, who is now 18, signed her informed consent for publishing the current case vignette.

## Case Vignette

The patient was a 17-year-old female (for the sake of convenience and literary style, the patient is referred to as “Jane” from now onwards). Her childhood history has included speech therapy since the age of 3 years. Multiple dyslalia and sensory integration difficulties were diagnosed at the age of six. Her intelligence quotient was within the normal range and she had no problems with school performance. Nevertheless, social adjustment problems were observed in her peer group.

Firstly, mood symptoms had occurred 2 years before index admission. Following a suicide attempt 3 months later, sertraline 50 mg/day had been started with no effect. Jane was admitted to the Adolescent Psychiatry Ward the following month due to suicidal tendencies. These included thoughts and ruminations on how to commit suicide along with visions/daydreams of seeing herself already dead. Self-injurious behavior was also present, namely scratching her skin with fingernails. The girl showed various psychotic symptoms after being admitted such as feelings of being persecuted and auditory, visual, and cenesthetic hallucinations. A diagnosis of paranoid schizophrenia was established according to the ICD-10 classification. The following treatment was administered sequentially: lorazepam + olanzapine, olanzapine + perazine, aripiprazole, perazine + opipramole, but to no avail. Improvement was achieved after introducing clozapine at 200 mg/day. The treatment was continued in an outpatient setting with clozapine combined with aripiprazole; however, full remission was not achieved.

At the end of 2019, the patient was again admitted to hospital. Increasing the clozapine dose was limited by side effects occurring, and introducing risperidone and lorazepam as an add-on maneuver did not improve the mental state. Jane was then qualified for ECT. There were no contraindications to ECT following neurological consultation, brain MRI, EEG, internal medicine consultation, and jugular artery Doppler ultrasound, in accordance with our hospital's standard procedure. Lorazepam withdrawal was then started. A date was planned for the first ECT session.

Four days before the first ECT, Jane had a generalized epileptic seizure with tongue biting and urination. The ECT was thus postponed and a slower withdrawal of lorazepam was recommended. No additional treatment was initiated due to the absence of any history of epilepsy and because of an incident during benzodiazepine withdrawal. An EEG was conducted and besides quite numerous delta waves in the frontal regions, which had been seen before, several short, generalized discharges of sharp and slow waves were recorded in the right hemisphere of the brain. The patient was treated with clozapine and EEG abnormalities, such as slow waves and epileptiform discharges, were quite often observed (5).

Nevertheless, the patient experienced a short blackout 10 days later with anterograde amnesia and she then fell over the next day. When staff arrived, she did not respond despite her being conscious. Neurological consultation was conducted and treatments were proposed; however, she suffered from another seizure a few hours later. Initially, there was poor rapport; nonetheless, 15 min later, Jane was able to swallow administered drugs, but convulsions started about a minute later. Diazepam was then administered intravenously, 5 mg, followed by a 10-mg dose. After the convulsions had been resolved, Jane did not regain consciousness and was then transferred to the neurological ward. During the transfer, the patients' condition started to improve.

Jane was sleepy on admission to the neurological department and did not respond to questions nor react to commands; her pupils were equal, round, and poorly reactive to light and accommodation. Meningeal or pyramidal signs were not detected during neurological examination and muscle tension was decreased, probably due to prior diazepam administration. Valproic acid was then introduced, first intravenously and then per os, because consciousness was still impaired after diazepam administration.

Valproic acid was introduced, first intravenously and then per os. EEG revealed generalized delta waves and a diffused slowing without evident seizure activity.

Several diagnostic procedures were carried out to exclude any organic causes of seizures. Brain contrast MRI was normal. Lumbar puncture was performed with normal cerebrospinal fluid (CSF) findings. Borreliosis, neuroborreliosis, HSV CNS infection, and Wilson's disease were excluded while CSF cultures were negative.

Laboratory tests also showed no abnormalities in liver, pancreas, and kidney function. Ammonia, lactate, and thyroid hormone levels were normal. Anti-TPO, anti-TSH, and anti-ATG antibodies were absent. Screening tests turned out negative for rheumatic diseases, including those for erythrocyte sedimentation rate, lupus anticoagulant, rheumatoid factors, and antinuclear and anti-neutrophil cytoplasmic antibodies. Although there was no medical history of streptococcal infections, the measured antistreptolysin O level was negative. Finally, no oligoclonal bands nor anti-neuronal antibodies were found in the CSF as an autoimmune etiology had also been considered.

During hospitalization, Jane experienced a seizure, which was interpreted as a psychogenic incident; the patient was laying down with eyes closed, breathing deeply, did not respond to questions or commands, and demonstrated irregular left upper limb lifting and lowering movements of the shoulder. The incident lasted 10 min and was stopped on saline administration. Further epileptic seizures or psychogenic incidents were not detected during video-EEG, which was afterwards performed.

Autoimmune encephalitis was considered (due to the coexistence of seizures and psychiatric disorders), although the course of disease was not typical. Due to EEG results, encephalopathy was considered in differential diagnosis, but eventually the most probable cause of EEG abnormalities and seizures was clozapine administration along with benzodiazepines being withdrawn. The psychogenic incident had made the final diagnosis more difficult.

The patient's clozapine dose was reduced and zuclopenthixol (5 mg a day) added after re-transfer to the adolescent psychiatry ward; there was no improvement. Multiple-type delusions, hallucinations, seizure-like events, posturing, and food restriction persisted. The EEG showed persistent slow waves in the frontal regions bilaterally, but focal sharp waves were not observed in the right temporal region. No full seizures were observed despite the presence of the seizure-like events. A team of experts (high-level specialists in psychiatry, ECT, child and adolescent psychiatry and neurology) thus decided to introduce ECT due to the patient's life-threatening condition and lack of any other treatment options, despite the contraindications. The legal guardians (parents) were fully informed and signed their informed consent, as required by Polish law. Jane was unable to participate in the decision process; nevertheless, she consented to the treatment protocol. The following treatment protocol was introduced: lorazepam and valproate withdrawal within a 2-week period, and in case of seizure, then an emergency administration of diazepam or midazolam, clozapine dose at 125 mg/day with EEG every 7 days. Withdrawing the drugs to increase the seizure threshold before ECT is highly recommended, although dose tapering may also be considered. Withdrawal of valproate and benzodiazepines was associated with some risk of seizures' recurrence. Nevertheless, this treatment protocol was introduced because epilepsy had not yet been diagnosed at this point.

Continuing on with benzodiazepines and flumazenil administration, just before the ECT, could also be recommended according to the literature (6). This is, however, not standard practice in Poland, where using flumazenil is generally limited to severe cases of intoxication to benzodiazepines.

There was no full seizure during this period; however, benzodiazepines were administered on five occasions. While benzodiazepines were being given as required, the patient was standing, trembling, looking forward, not responding to questions nor comments, and her muscle tone was increased.

As before, a date was settled for the first ECT, when a sudden high temperature and respiratory distress occurred. The ECT was thus canceled once more. Jane was then transferred to the intensive care unit and diagnosed with (probably aspiration) pneumonia followed by treatment. The patient was re-admitted to the psychiatry ward 6 days later. Her mental state was even worse than before at readmission. The girl was alert, hypokinetic, mute, and unable to undertake even basic activities where she was fed by feeding tube. At this point, catatonia syndrome became fully developed (as we believe associated with schizophrenia), for which elective treatment includes ECT. Thus, the previous treatment plan was implemented and an ECT protocol was finally started the next day.

Within 12 weeks, 24 ECT sessions had been delivered. Bilateral electrode placement was used. The seizure duration for each of the 24 treatments was adequate, lasting at least 20 s. Doses of anesthetics used were atropine (0.3–0.5 mg), thiopental (250–300 mg), and succinylcholine (70–80 mg).

Neither seizures nor any other complications were observed during this time. Over 3 months, 24 ECT sessions had been delivered at an average rate of twice weekly. The feeding tube could be removed after the first ECT session and her further mental state gradually improved although residual symptoms were still present like flattening effect, reduced insight, and residual psychotic symptoms. Despite the obvious improvement, the patient's constant complaint was suicidal ideation. She reported suicidal thoughts every morning to her physician but afterwards remained active and cheerful during the rest of the day. The discrepancy observed between definite improvements to her mental state by the treatment team and the unchanged severity of the suicidality reported by Jane was discussed within the treatment team and with the external supervisor of psychodynamic theoretical orientation. The behavior was interpreted in the context of the forthcoming discharge. Further steps including continuing treatment in a long-term therapeutic unit were discussed with the patient. After the ECT protocol had been completed, the clozapine dose was increased up to 175 mg a day and 3 days later the patient was released from the hospital. According to the treatment plan, she was then admitted to a therapeutic unit, focused on sociotherapy, family therapy, and psychotherapy as well as educational rehabilitation. According to the literature, ECT maintenance should be considered. This option seemed somewhat unpromising, as the effectiveness of the administered ECT sessions became lowered over time despite charge increase; seizures lasted shorter and any improvement in patient's behavior, psychotic symptoms, and suicidal tendencies had diminished when compared with the outcomes of the first ECT sessions.

The treatment team believed psychiatric rehabilitation was crucial over this treatment phase. Moreover, we had to take into account numerous administrative issues and obstacles (discussing the problems of our mental health system lies outside the scope of our paper). Summing up, the benefits did not exceed the disadvantages of this treatment option.

Jane stayed in the aforementioned unit until February 2021. Her functioning within her peer group was satisfactory according to the unit's staff (Ł.S.); she had fully engaged at school and the therapeutic program. Positive symptoms were present almost every day (mainly imperative and commenting voices); nevertheless, the patient was able to cope with them. Her mood was stable and the suicidality was not observed. A clozapine dose of 375 mg/day, hydroxyzine 25 mg, or levomepromazine 25 mg was administered *ad hoc* whenever needed.

The patient presented with paroxysmal incidents in the therapeutic unit, which were first recognized to be associated whenever hallucinations became intensified, following her subsequent explanations. During such episodes, the patient froze-up stiff, with limbs shaking, and she was unresponsive to either questions or commands; sometimes on just freezing-up stiff, with subsequent falls, or during temporary lapses of awareness, she was staring or her muscles became stiff. The paroxysmal episodes increased in frequency, despite modifying her psychiatric treatment. Increased frequency of episodes was not associated with the patient's mental state deteriorating. Although the recurrence of catatonia could be taken into consideration in differential diagnosis, medical interventions were thereby focused on verifying whether epilepsy was suspected. An EEG demonstrated paroxysmal discharges of spike and wave complexes in the right hemisphere and sharp-and-slow wave complexes in the left temporal region. During another EEG, the following clinical incident was observed by the performing technician: first, a shaking of the right limbs, then head turning to the right and a bending forward of the upper body. Jane did not respond to questions nor commands throughout this time; however, afterwards, her responsiveness became normal. Unfortunately, any bioelectrical assessment of the recorded EEG was not possible due to technical artifacts. Lamotrigine was given as epilepsy was suspected and the patient was then referred to the neurological ward. Jane described these aforementioned episodes during her hospitalization as being preceded by an undefined feeling of a seizure approaching and the limb shaking and during the event as being associated with impaired consciousness. Her mother had witnessed most of such incidents and reported episodes of seeing her daughter freezing-up stiff with an unnatural positioning of the hands and feet, followed by limb shaking (and squeezing objects that are held in hands), skin pallor, and hyperventilation. During such episodes, the patient was unresponsive to questions or commands and if she moved forward, she tripped over and fell. The incidents lasted 1–2 min and postictal confusion was absent. After introducing lamotrigine, such episodes substantially decreased from 10–15 to 2–5 episodes per day. Neurological examination showed a slight extrapyramidal muscle tone and intention tremor to be present. Hippocampal asymmetry was visible (*P* < L) on brain MRI without focal lesions. An EEG was abnormal with recurring delta waves in the temporal bilateral regions, and there were paroxysmal discharges of spike and wave complexes in the left hemisphere and right temporal region. During long-term video monitoring, Jane reported experiencing several “seizures” of auditory hallucinations and an accompanying anxiety. She sometimes felt that she had had a seizure without remembering the actual details. No paroxysmal activity was detected in EEG during any of the episodes. These paroxysmal incidents so described were clinically untypical for epileptic seizures; the exacerbating hallucinations causing the freezing-up or catatonic symptoms were considered. However, a favorable response to lamotrigine during paroxysmal incidents does not necessarily prove their epileptic etiology. Although there is scant evidence on the efficacy of lamotrigine in treating anxiety, any positive effect cannot be ruled out. The episode observed during EEG was suspected to be epileptic, so lamotrigine had been continued.

According to Jane, she still considers the decision for her starting ECT therapy, the one year previous, to have been right. The main justification was an insufficient effect of psychopharmacotherapy with there being no possibility of increasing doses. The main difficulties encountered with the ECT therapy were a long hospital stay and separation from the family (due to the COVID-19 pandemic, there were no home passes and no visits allowed). She considered that nuisances associated with the treatments themselves to be less important (i.e., preparation for ECT and memory impairment). The patient was also satisfied with the implemented pharmacotherapy.

Introducing ECT was believed by the treatment team to be beneficial and safe for the patient. The diagnosis of epilepsy may be considered a complication of the previous ECT. However, this may also not be so. The first seizure occurred before introducing ECT. Moreover, the patient was and is still using clozapine, which is associated with an average 3% seizure risk (7). Notwithstanding, the patients' life was in the imminent danger and no other treatment option was then left open except for the ECT. Timeline is summarized in Table 1. ECT parameters summary is presented in Table 2.

**Table 1:** Sequence of events.

Age 15 y 4 months	First symptoms: anhedonia, lower energy level, depressed mood
15 y 6 m	Feeling empty inside and numb
15 y 7 m	Self-mutilation started
15 y 8 m	Suicide attempt (swallowing washing capsule)
15 y 8 m	First psychiatric consultation, outpatient setting, sertraline 50 mg/s started
15y 9 m – till 16 y 0 m	Hospital admission due to suicidal ideation. Diagnosis of schizophrenia established. Pharmacotherapy, dose upgrading was limited due to side effects: Olanzapine up to 15 mg/d + lorazepam up to 3 mg/d Olanzapine up to 15 mg/d + perazine up to 100 mg/d Aripiprazole up to 15 mg Perazine up to 500 mg/d Clozapine 200 mg/d – improvement and discharge
16 y 2 m	Relapse. Increasing clozapine up to 300 mg/d in outpatient setting
16 y 6 m	Clozapine + aripiprazole up to 15 mg/d; improvement, residual auditory hallucinations persisted
17 y 4 m	Admitted to hospital due to psychotic symptoms, depressive mood, suicidal plans and food intake restriction
17 y 4 m – 17 y 5 m	Inpatient treatment with no improvement: Clozapine dose increase up to 375 mg/d, further up-dosing limited by side effects Clozapine + risperidone up to 2 mg/d
17y 6 m	Qualified for ECT Standard precautions^a^ undertaken: brain MRI, EEG, jugular vein doppler ultrasound, neurological consultation, consultation of internal medicine – no contraindications revealed Lorazepam withdrawal started First ECT session scheduled for 31th Dec
17y 6 m	Generalized epileptic seizure with tongue biting and urination
Next ten days	Slower lorazepam withdrawal recommended EEG was conducted ECT session postponement
Next day	Blackout with anterograde amnesia
Next day	Faintness, lack of responsivity, seizure-like state – transfer to the Intensive Neurological Treatment Unit. Extensive laboratory testing, valproic acid introduced
After ten days	Retransfer to the psychiatric unit Clozapine continued and zuclopentixol 5 mg/d added with no effect
After two weeks	After experts' team discussion qualified to ECT again
Next two weeks	Lorazepam gradual withdrawal Valproic acid gradual withdrawal Clozapine 125 mg/d EEG conducted every 7 days At 5 occasions due to seizure-like events benzodiazepines were administered *ad hoc* No full seizure occurred
Day 0	First ECT session scheduled. Before the session severe pneumonia symptoms occurred.
	Transfer to the pediatric Intensive Treatment Unit.
Six days later	Readmission to the psychiatric unit
Next three months	24 ECT sessions. Clozapine dose 125 mg/d No severe adverse events occurred.
Three days after the last ECT Just before 18th birthday	Discharged from the hospital. Treatment continued in the long-term therapeutic unit.
Next 7 months	Treatment continued in the therapeutic unit.
18 y 8 m	Lamotrigine introduction Admission to neurology ward. Diagnosis of epilepsy with partial seizures established. Co-occurrence of non-epileptic seizure-like events confirmed.

*ECT, electroconvulsive therapy; EEG, electroencephalography; MRI, magnetic resonance imaging*.

a *precautions include also electrocardiography which in this case had been already conducted*.

**Table 2:** ECT charge and duration.

**ECT session number**	**Charge** **(100% = 504mC)**	**Pulse width** **(per minute)**	**Seizure duration (seconds)**
1	25%	68–76	45
2	30%	76–86	69
3	30%	69–93	60
4	30%	60–75	65
5	30%	70–80	55
6	35%	96–96	40
7	45%	65–91	55
8	45%	82–92	41
9	55%	81–82	45
10	65%	110–114	44
11	75%	86–125	35
12	90%	49–85	40
13	100%	81–87	27
14	120%	81–107	20
15	140%	81–110	40
16	160%	90–120	24
17	180%	65–76	20
18	200%	90–120	22
19	200%	73–93	28
20	200%	83–98	20
21	200%	78–100	20

## Discussion

### Electroconvulsive Therapy in Adolescents

ECT in children and adolescents is believed to be a safe and effective treatment method (8). The American Academy of Child and Adolescent Psychiatry (AACAP) guideline includes three criteria for patients qualifying for ECT:

(1) Diagnosis—severe depression and manic episodes with or without psychotic features, schizoaffective disorder, schizophrenia, catatonia (in some cases the treatment of choice), and neuroleptic malignant syndrome.
(2) Severity of symptoms—intensity of symptoms must be severe, persistent and life threatening.
(3) Lack of treatment response to appropriate psychopharmacological agents accompanied by other appropriate treatment modalities. ECT may be considered earlier in cases of:
  (a) Intolerability of psychopharmacological treatment.
  (b) The adolescent being unable to take medication due to incapacity.
  (c) Waiting for a response to a psychopharmacological treatment may endanger the life of the adolescent.

The AACAP does not point out any absolute medical contraindications for ECT in adolescents. Relative contraindications include central nervous system tumors with increased cerebrospinal fluid pressure, recent myocardial infarction, and active pulmonary infection (9).

Although not enlisted in the aforementioned paper, the use of ECT in psychiatric patients with comorbid epilepsy may be considered a serious clinical challenge (10, 11).

### Electroconvulsive Therapy and Epilepsy

Although the ECT mechanism of action is directly related to the ictal activity of the brain, this procedure is not related to epilepsy as understood as being a chronic neurological disease (12). The basic mechanism of ECT is to intentionally induce an electroclinical seizure. ECT may be difficult to induce in individuals treated for epilepsy because most are on anti-epileptic medications. Evidence-based knowledge is sparse about the advantages and disadvantages of combining antiepileptic drugs with ECT, and guidelines on this have been contradictory. It is believed that in some cases dose reductions are required. There have also been concerns that after ECT, the incidence of epilepsy may increase. This was, for example, recently observed in a Danish cohort study of adults with affective disorders, for an under 40s age group (13). The question remains whether this was the effect of ECT or of other confounding factors associated both with epilepsy and psychiatric disorders.

Still, clinical experience shows that the aforementioned fears concerning the use of ECT are unfounded in patients with psychiatric disorders who also suffer from epilepsy. The seizure threshold remains relatively stable during electroconvulsive therapy or—more often—constantly increases. Indeed, it has been empirically proven (i.e., 10) that ECT results in reduced psychiatric symptoms; moreover, some patients appear to demonstrate markedly reduced rates of spontaneous seizure after completing their ECT course. Therefore, one can conclude that most epileptic patients can be treated with ECT without adjusting the doses of antiepileptic medications and that ECT can be recommended for safe use in cases of patients with psychiatric disorders with comorbid epilepsy.

ECT in minors with comorbid epilepsy was discussed by Maixner (6) who stated that the procedure is safe. If discontinuing the antiepileptic drugs is not possible, tapering the dose and a 12-h washout before each ECT is recommended. These guidelines were based on the previous literature, which includes a case series of 43 adult patients with epilepsy successfully treated with ECT for comorbid psychiatric disorders (10). A few case studies have been published on children that focus on treatment-refractory epilepsy, although the routine use of ECT for refractory status epilepticus is not recommended (14). Case descriptions included also ECT in minor with anti-NMDA encephalitis associated with catatonia (15). There are almost no descriptions in the literature of ECT being psychiatrically indicated in adolescents with seizures. One case study (16) has referred to a 17-year-old male adolescent with depression, myelomeningocele, and about once a year seizure. A study on ECT in 20 adolescent patients included a female where epilepsy had not worsened and who had a long-lasting seizure disorder and psychiatric illness. Another patient did not exhibit further complications who had had one grand-mal seizure just before the ECT course (17). A further study mentions a favorable outcome of ECT therapy despite drug-induced seizure during the treatment course (18). Diagnoses were, however, not provided in these aforementioned cases. A young adult female patient with schizophrenia (19) had also presented with a condition similar to the one described herein that included clozapine-induced seizure and a favorable ECT course.

### Case Discussion

We believe that due to the clozapine inefficacy and severe illness course in our patient, the first decision for introducing the ECT procedure was in line with experts' recommendations and could be considered as a treatment of choice both in adults and in adolescents. Also, the combination of clozapine and ECT is widely approved (20).

Of course, when analyzing the treatment options, the first step was establishing a diagnosis. Multiple delusions included ideas of persecution, reference, thought insertion, guilt, and catastrophe. Reported hallucinations were auditory (e.g., simple sounds, whispers, numerous imperative and commenting voices), visual (e.g., blood, a dead body, a cat), olfactory (smoke), taste (altered food flavor), and coenesthetic (feeling the ground shaking or a fire inside the body). The rapport with her was poor, eye contact was poor, and affect was flat. Thus, the symptom profile in the differential diagnosis included psychotic depression. Our treatment decisions were to be based on the ICD-10 classification due to administrative regulations and the catatonia syndrome should not be analyzed per se, but should be considered as either depressive stupor or catatonic schizophrenia.

Mood symptoms are not uncommon in the prodromal phase of psychosis (21). Nevertheless, after full-blown psychotic symptoms have developed, the distress is attributed to the very unpleasant psychotic symptoms. Although these symptoms may be interpreted as being depressive-mood congruent, we believed the deteriorated mood to be secondary to terrifying and psychotic experiences. Also, suicidal ideation, reported by the patient almost all the time, was not accompanied by an affective component. Thus, the clinical picture corresponded more with schizophrenia than affective or schizoaffective disorders, although the latter possibility would be verified during a long-term course of the illness.

Catatonic symptoms, namely posturing and mutism, were observed occasionally throughout with varying severity. Also, seizure-like states, which emerged after the full seizure occurred, may be considered as being catatonic symptoms. It was not possible to definitely establish the symptoms' nature due to complex clinical picture (this diagnostic problem still remains unresolved). Finally, just before the first ECT and after the pneumonia had resolved, a full clinical picture of catatonia was present and quickly resolved after introducing the actual ECT.

Summing up, the diagnosis of paranoid schizophrenia was sustained after taking into consideration the whole illness course. Nevertheless, mood symptoms seen at the beginning of the illness beginning, as well as catatonia traits, were considered factors that increased the likelihood of a positive ECT treatment effect. However, epileptic comorbidity occurred during the preparation phase and lorazepam withdrawal, where the severity of seizures required transfer to the intensive treatment unit. This was the reason why the treatment options had to be reconsidered.

After an in-depth case discussion, the experts' consylium concluded that the seizures seemed to be associated with the clozapine treatment and modifications to the drugs' doses. Indeed, neither during nor after the ECT course was anti-epileptic treatment necessary. Unfortunately, in longer-term observation, the paroxysmal event frequency increased. Although the epilepsy diagnosis is now established, not all paroxysmal events seem to be epileptic in their nature.

At the moment of ECT introduction, the patient's life was in imminent danger and no other treatment option was then left open except for the ECT. We thus still believe this decision to have been optimal under such circumstances.

### Strengths and Limitations of the Study

Early-onset schizophrenia (EOS; the schizophrenia age of onset <18) is a rare and more severe form of the disease. There is a dearth of studies regarding treatment options for such a condition. Recommendations are either limited to the most basic or based on the experts' consensuses when extrapolating results from adult studies. We thus believe the subject that our study is focused on to be important. We also had the fortune of working in a multidisciplinary hospital with multiple diagnostic possibilities. This enabled us to consider the complexity of the patient's condition from both the neurological and psychiatric point of view. Nonetheless, the dearth of previous data is also a limitation of the study as we are not able to refer to previous research.

Another study limitation is diagnostic difficulties regarding the seizures' characteristics. Co-occurrence of epileptic and epileptic-like events is not an uncommon phenomenon (22) and was also experienced by our patient. The situation also made assessment of the ECT treatment risk/benefit ratio less certain. Moreover, the diagnosis of epilepsy at follow-up makes the outcome not so favorable.

## Conclusion

ECT is a treatment option that may be effective in life-threatening conditions. Seizures and epilepsy are not absolute contraindications. Studies to date do not exclude the possibility of an increased risk of epilepsy developing after ECT; however, results are inconsistent. In our case, we managed to confirm a diagnosis of epilepsy within a few months following the ECT course. Because the first seizure occurred before giving the ECT, other etiological or confounding factors, including pharmacotherapy, should also be considered.

The ECT in combination with clozapine turned out to be effective and not associated with any imminent complications; the recovery course indicates the significance of psychosocial interventions even in those adolescents who are endogenously severely ill. We believe that the introduction of ECT was necessary in our case due to life-saving indications. The follow-up neurological diagnosis shows that further studies and more data are required on this treatment procedure in minors.

## Data Availability Statement

The original contributions presented in the study are included in the article/supplementary material, further inquiries can be directed to the corresponding author/s.

## Ethics Statement

Written informed consent was obtained from the individual(s) for the publication of any potentially identifiable images or data included in this article.

## Author Contributions

AG: case description, litarature search regarding ECT in adolescents. BR: study design, general literature search, and preparing final version of the manuscript. ŁS: case description regarding current patient functioning. IK-J: literature search and case description regarding EEG results. MK and HS-J: case description regarding the neurological diagnosis and treatment. AA-W and ŁŚ: literature search focused on ECT and epilepsy, preparing this part of the manuscript. All authors contributed to the article and approved the submitted version.

## Funding

The publication has been supported by Angelini Pharma Polska Sp. z o.o. The funder was not involved in the study design, collection, analysis, interpretation of data, the writing of this article or the decision to submit it for publication.

## Conflict of Interest

The authors declare that the research was conducted in the absence of any commercial or financial relationships that could be construed as a potential conflict of interest.

## Publisher's Note

All claims expressed in this article are solely those of the authors and do not necessarily represent those of their affiliated organizations, or those of the publisher, the editors and the reviewers. Any product that may be evaluated in this article, or claim that may be made by its manufacturer, is not guaranteed or endorsed by the publisher.
